# Go Away from Home to Learn the News

**Published:** 1881-05

**Authors:** 


### GO AWAY FROM HOME TO LEARN THE NEWS.

The efficacy of nitrite of amyl appears so well demonstrated in these cases (chloroform poisoning), that in America they compel the dentists, who in that country use and abuse chloroform, to always have the *nitrite* at hand in case of accident (!)—*Journal de Medecine et de Chirurgie*, January, 1881.—W. W. S.

Since when ?

